# Dynamics of fungal bioaerosols in Kuwait City: An 8-year survey

**DOI:** 10.1371/journal.pone.0329318

**Published:** 2025-08-13

**Authors:** Abdullah AlSaleh, Mai Marafie, Hasan Dashti, Laila Ashkanani

**Affiliations:** 1 Microbiology, Immunology and Infectious Diseases Dept., College of Medicine and Health Sciences, Arabian Gulf University, Manama, Bahrain; 2 Occupational Health Directorate, Ministry of Health, Kuwait City, Kuwait; 3 Department of Meteorology, Directorate General of Civil Aviation, Kuwait City, Kuwait; SKUMS: Shahrekord University of Medical Science, IRAN, ISLAMIC REPUBLIC OF

## Abstract

Fungi are ubiquitous eukaryotic microorganisms that are often implicated in a wide range of diseases. Exposure to fungal bioaerosols is often associated with conditions such as asthma, allergic rhinitis, keratitis, and pneumonitis, among others. This study aims to shed some light on the configuration of airborne culturable fungal genera in the atmosphere of Kuwait City with analysis of seasonal patterns, intra-diurnal variations and correlations with meteorological factors. Outdoor fungal bioaerosols samples were collected twice a day, two times a week, from January 2016 to December 2023 via volumetric active sampling in Al-Sabah Medical District, Kuwait City, Kuwait. The meteorological data were supplied by Kuwait Meteorological Department, Directorate General of Civil Aviation. The rates of fungal bioaerosols remained relatively high during the months of March, April and May, ranging from 269 to 296 CFU/m^3^. *Cladosporium*, *Aspergillus* and *Alternaria* were the most common fungal genera in the atmosphere, with a prevalence of 49%, 16% and 14%, respectively. Statistically significant differences (p < 0.05) in fungal composition between intra-diurnal periods were demonstrated in most monthly analyses. A strong negative correlation was demonstrated with temperature and haze events. Conversely, positive correlations were observed with relative humidity, wind action, rainfall and dust phenotypes. Fungal dynamics in relation to seasonality and intra-diurnal variations require further elucidation. Collaborative research is needed to advance our understanding of the implications of elevated rates of fungal bioaerosols, the seasonality of specific fungal allergens and what may be suitable means of mitigation. Efforts such as this study will hopefully help facilitate regionally specific air quality management decisions and provide baseline information for health-care providers.

## Introduction

Fungi are ubiquitous eukaryotic microorganisms that are often implicated in a wide range of diseases [1]. Exposure to fungal bioaerosols is often associated with conditions such as asthma, allergic rhinitis, keratitis, and pneumonitis, among others [2]. They are small in size and quantitively abundant in the atmosphere and have the potential to infiltrate indoor environments [3]. Fungal conidia are usually less than 10 µm in size and are able to reach the lower respiratory tract as well as the nasal and paranasal sinuses, where mycotic inflammation may ensue [4,5]. The distribution of these fungal spores is often analyzed via aerobiological volumetric methods, which enable the determination of seasonal conidial concentrations as well as spore emission peaks throughout the day [6]. These investigations are made possible by utilizing volumetric active sampling instruments that use pumps (artificial force) to control airflow through the sampler to collect fungal spores from the atmosphere [7]. Depending on the model of the instrument and the power of the pump, a specific volume of air can be sampled [7]. This allows the results to be expressed in concentration (CFU/m^3^); thus, clinical and environmental comparative investigations can be conducted [7]. These investigations assist in correlating environmental events with illness in people suffering from fungal allergens [6].

Moreover, atopic and immunocompromised individuals are at risk of developing acute hypersensitivity reactions following exposure to fungal spores [4,8]. In fact, high aerial counts of fungal conidia are significantly associated with asthma-related emergency clinic visits [9,10]. Several environmental and meteorological factors may influence the distribution and configuration of fungal bioaerosols in the atmosphere; factors such as relative humidity, wind velocity and dust storms often increase fungal dispersal, whereas high temperature, rainfall and precipitation are often perceived as limiting factors [8,11–14].

Kuwait is a developed, middle eastern, metropolitan country located in the Arabian Peninsula. Its geographical location provides certain challenges, such as low topography, scant vegetation, strong winds, and low annual precipitation rates, with surrounding military conflicts contributing to more desertification in the area [15,16]. Consequently, frequent dusty weather is a common phenomenon; in fact, the monthly average dust concentration in Kuwait is one of the highest in the world, reaching up to 1400 µg/m^3^ [17]. Additionally, the average dust deposition rate in Kuwait has been recorded to be 0.59 kg/m^2^ annually, ranging from fine to very fine sand fractions [18]. Alarmingly, soil and dust deposits in Kuwait may harbor potentially pathogenic bacteria such as *Mycobacterium*, *Coxiella burnetii*, *Clostridium perfringens*, and *Brucella* [19]. In addition to dust, elevated levels of fungal bioaerosols have been reported in Kuwait, reaching a high rate of 2.66x10^6^ cells/m^3^, which is comparable with the high levels reported in tropical regions [12,17]. Indeed, the respirable contaminants in Kuwait’s atmosphere present a critical challenge for healthcare providers, as the estimated mortality burden related to respirable aerosols is 52.3 per year [20]. Moreover, the reported high rate of sensitization to fungi in asthmatics in Kuwait is implicated in triggering exacerbation episodes [4]. Thus, there is a pressing need to investigate potentially pathogenic respirable bioaerosols suspended in Kuwait’s atmosphere. Therefore, this study aims to shed some light on the configuration of airborne culturable fungal genera in the atmosphere of Kuwait City with analysis of seasonal patterns, intra-diurnal variations and correlations with meteorological factors.

## Materials and methods

### Location

The current study was carried out from January 2016 to December 2023 (excluding April and May 2020 due to the COVID-19-related lockdown) in Al-Sabah Medical District (SMD), which is located on the western side of Kuwait City, Kuwait. SMD is host to more than 17 hospitals and medical centers as well as universities, applied education colleges, research institutions and medical staff housing. The specific sampling location was outside the Al-Rashed Allergy Center at 29° 21’ 52.2’‘ N, 47° 59’ 20.04’‘ E.

### Sampling method

Volumetric active sampling via the impactor Andersen viable particle sampler (ThermoScientific, USA) was used in this study. The stage contains 200 tapered orifices 0.4 mm in diameter (respirable), with constant airflow at 28.3 liters/minutes. The recommended height during sampling was 100–150 cm above ground to simulate the average human breathing zone, our study was conducted at a fixed height of 120 cm. To avoid plate overgrowth, each sample consisted of 85 L of air, as recommended by the manufacturer and the aerobiology department standards. The instrument was carefully disinfected with 70% ethanol after each sample.

Two air samples were taken at 7:00 am and 1:00 pm twice a week throughout the study period. All samples were taken in duplicate. The average concentration (CFU/m^3^) of the four samples was used to represent daily values. The samples were collected on potato dextrose agar (PDA) CM0139 (Oxoid Ltd., UK). The plates were incubated for 5 days at 25°C, and the growing fungi were examined under a microscope for morphological identification. Fungal colonies were converted into colony-forming units per cubic meter of air (CFU/m3) via the following formula:


$$Concentration\left(CFU/m^{3}\right)=\frac{NumberofColonies(n)X1000}{FlowRate(Liters/minutes)}\times SampleDuration(minutes)$$

### Meteorological Parameters

Meteorological data, including maximum, minimum, and mean temperatures (T_max_, T_min_, T_mean_), expressed as °C, relative humidity (%), rainfall (mm), wind speed (m/s) and wind speed (m/s), among others, were supplied by Kuwait Meteorological Department, Directorate General of Civil Aviation, the official source of weather information in the country. Meteorological data such as temperature, relative humidity and wind speed data were acquired from Kuwait City Station (29° 23’ 7.679’‘ N, 47° 58’ 48.338’‘ E), whereas rainfall, dust and haze data were collected from Kuwait International Airport Station (29° 14’ 26.126’‘ N, 47° 58’ 15.779’‘ E).

In accordance with the standards of the Department of Meteorology in the Directorate General of Civil Aviation of Kuwait, dust events were classified on the basis of visibility and wind speed according to the following three categories: [1] dust storms: visibility ≤ 1000 m and WS ≥ 8 m/s; [2] rising or blowing dust: visibility ≥ 1000 m and WS ≥ 8 m/s; and [3] dust haze: visibility ≤ 5000 m and WS ≤ 8 m/s [21].

### Permit statement

No specific permissions or permits were required for the publication of this work. All data and materials referenced herein are either publicly accessible at the aforementioned departments or originally created by the authors.

### Statistical analysis

Descriptive and inferential statistics were employed to analyze daily fungal spore counts. The Shapiro‒Wilk test was used to test the normality of the data distribution. Levene’s test was used to test the equality of variances. Independent t tests and Mann‒Whitney U tests were applied to compare the differences in means. One-way ANOVA and the Kruskal‒Wallis test were used for parametric and nonparametric analyses, respectively. A Bonferroni post hoc correction was employed for pairwise comparisons to identify specific variances that were significant among different groups. The weighted Jaccard similarity coefficient was used to compare the similarities in monthly fungal composition over the study period, as well as between the two intra-diurnal periods (am vs pm), following the formula below:


$$J_{\left(a,b\right)}=\frac{\Sigma \min(a,b)}{\Sigma \max\left(a,b\right)}$$

where:

J = weighted Jaccard similarity coefficient; a, b = any tested time periods.

Jaccard similarity coefficient ranges from 0 to 1; with 1 indicating that the two groups are identical, and 0 indicating that the two sets have no shared elements.

Pearson’s correlation coefficient (r) was calculated to study the correlation between fungal data and the meteorological parameters of the same period. The statistical hypotheses were tested at the level of alpha (significance level) equal to 0.05, and the difference between elements was considered significant when p < 0.05. Data management and analysis were carried out via Microsoft Excel 365.

## Results

### Monthly variations in airborne fungal populations

**Fig 1:**
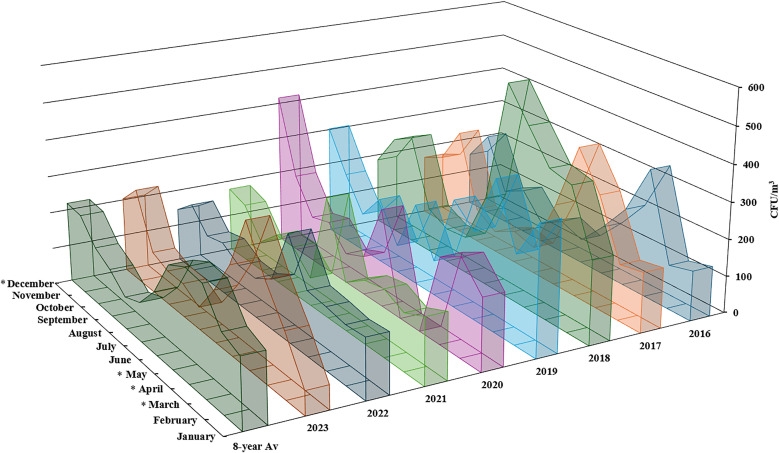
Monthly concentration (CFU/m^3^) of total fungal spore counts. Asterisks denote statistical significance (p < 0.05).

Outdoor fungal bioaerosol samples were collected twice a day, two times a week, from January 2016 to December 2023 (excluding April and May 2020 due to COVID-19-related lockdowns) in Al-Sabah Medical District, Kuwait City, Kuwait. Over the study period, the rates of fungal spores remained relatively high during the months of March, April and May, ranging from 269 to 296 CFU/m^3^, whereas in July and August, the lowest concentration was 86–106 CFU/m^3^, as shown in Table 1. Fungal spore rates mostly remained consistent throughout the study period, with the highest fungal bioaerosol concentration occurring in 2018 and the lowest fungal bioaerosol concentration occurring in 2016 (Fig 1). The highest recorded concentration was in May 2018, where it reached 577 CFU/m^3^, whereas the lowest concentration was 53 CFU/m^3^, which was recorded in August 2016 (Fig 1). The monthly mean spore concentrations in March, April, May and December were statistically significant (P < 0.05), as shown in Fig 1.

**Table 1:** Fungal spore concentrations (CFU/m^3^) collected from outdoor samples over 8 years. Data are presented as minimum-maximum (average). Data are presented in CFU/m^3^.

	January	February	March	April	May	June	July	August	September	October	November	December
*Alternaria*	9–32 (19)	9–156 (45)	31–52 (46)	29–76 (48)	39–105 (55)	12–56 (27)	9–57 (21)	3–19 (12)	4–24 (13)	4–17 (11)	5–33 (15)	9-32 (19)
*Aspergillus*	6–61 (31)	5–24 (16)	6–33 (19)	13–31 (23)	9–41 (19)	5–70 (43)	5–49 (20)	12–69 (28)	17–68 (37)	12–64 (48)	12–71 (41)	24–78 (41)
*Cladosporium*	26–188 (82)	43–179 (86)	87–263 (164)	52–374 (166)	38–349 (150)	26–117 (61)	18–61 (35)	9–45 (25)	35–72 (59)	25–156 (71)	90–155 (121)	51–341 (123)
*Drechslera*	0–4 (1)	0–7 (1)	0–5 (3)	0–9 (3)	0–10 (3)	0–4 (1)	0–4 (1)	0–4 (2)	0–4 (2)	0–4 (1)	0–10 (3)	0–3 (1)
*Mucorales*	0–7 (1)	0–3 (1)	0–5 (1)	0–1 (1)	0–3 (1)	0–8 (2)	0–21 (3)	0–3 (1)	0–2 (1)	0–2 (1)	0–3 (1)	0–3 (1)
*Penicillium*	8–53 (24)	12–49 (26)	13–40 (26)	10–25 (15)	7–10 (7)	0–26 (7)	1–11 (5)	1–5 (2)	3–12 (8)	2–14 (7)	4–19 (8)	11–35 (16)
Yeast	0–27 (8)	0–21 (5)	0–18 (5)	0–16 (9)	0–14 (6)	0–17 (6)	0–12 (3)	0–11 (3)	0–7 (3)	0–18 (4)	0–20 (5)	0–19 (5)
Total Fungi	64–351 (184)	119–390 (206)	167–406 (289)	169–490 (296)	130–577 (269)	66–324 (167)	61–220 (106)	53–131 (86)	79–178 (128)	83–303 (155)	159–272 (213)	145–416 (218)

**Fig 2:**
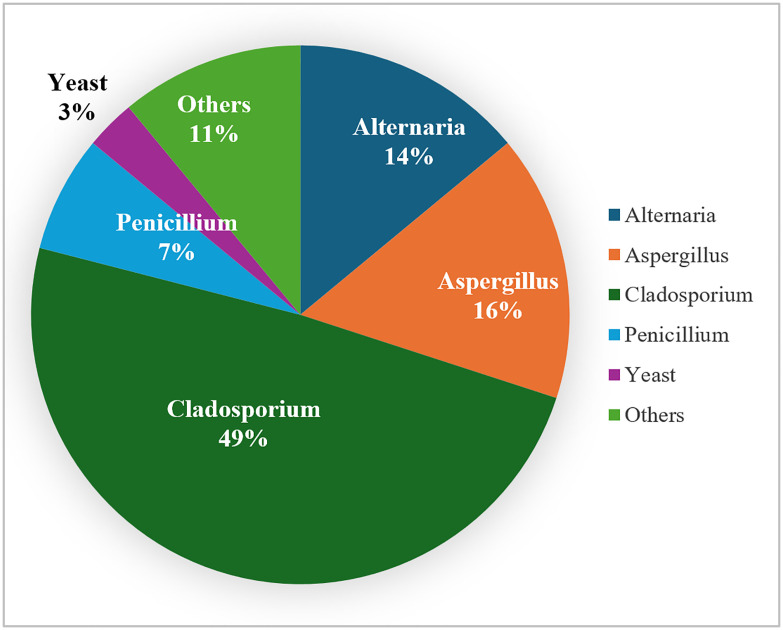
Composition of fungal genera in the atmosphere over the study period.

**Fig 3:**
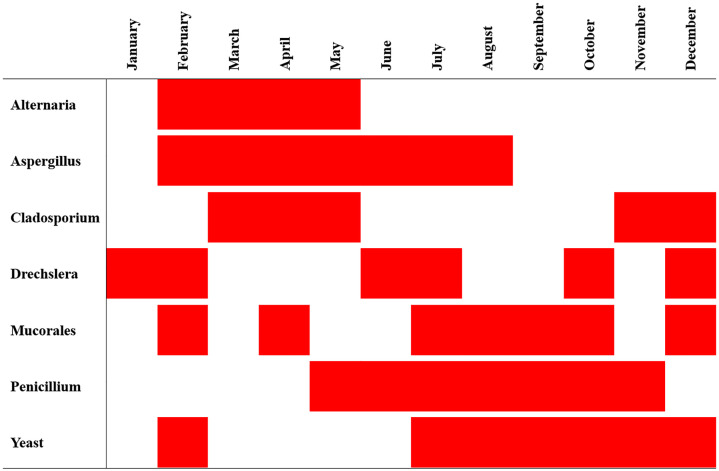
Statistically significant prevalence of airborne fungal genera per month during the study period. Statistical significance (P < 0.05) is denoted in red.

Moreover, *Cladosporium*, *Aspergillus* and *Alternaria* were the most common fungal genera in the atmosphere, with prevalences of 49%, 16% and 14%, respectively, as shown in Fig 2. Certain fungal genera followed statistically significant (P < 0.05) patterns of monthly distribution throughout the 8-year survey, as shown in Fig 3. *Alternaria* was significantly present in the atmosphere from February through May, while *Aspergillus* was significantly present for a longer period from February to August, and *Cladosporium* was split into two periods, March to May and November to December.

The highest Jaccard similarity coefficient score for atmospheric fungal composition was recorded between March, April and May and between November and December (Table 2). The lowest similarity coefficient was calculated between August and March (Table 2).

**Table 2:** Monthly similarity index with respect to daily fungal distribution. Similarity was determined through the weighted Jaccard similarity coefficient. Blue denotes maximal similarity, whereas red denotes minimal similarity.

Month	January	February	March	April	May	June	July	August	September	October	November	December
January	1.00	0.75	0.54	0.53	0.50	0.67	0.50	0.43	0.61	0.69	0.66	0.71
February	0.75	1.00	0.68	0.61	0.62	0.56	0.45	0.31	0.45	0.52	0.55	0.60
March	0.54	0.68	1.00	0.92	0.84	0.42	0.32	0.23	0.34	0.39	0.60	0.64
April	0.53	0.61	0.92	1.00	0.86	0.44	0.33	0.25	0.35	0.41	0.62	0.66
May	0.50	0.62	0.84	0.86	1.00	0.46	0.35	0.26	0.36	0.42	0.65	0.64
June	0.67	0.56	0.42	0.44	0.46	1.00	0.59	0.49	0.74	0.79	0.62	0.62
July	0.50	0.45	0.32	0.33	0.35	0.59	1.00	0.65	0.63	0.50	0.40	0.40
August	0.43	0.31	0.23	0.25	0.26	0.49	0.65	1.00	0.65	0.49	0.38	0.35
September	0.61	0.45	0.34	0.35	0.36	0.74	0.63	0.65	1.00	0.74	0.58	0.54
October	0.69	0.52	0.39	0.41	0.42	0.79	0.50	0.49	0.74	1.00	0.68	0.64
November	0.66	0.55	0.60	0.62	0.65	0.62	0.40	0.38	0.58	0.68	1.00	0.92
December	0.71	0.60	0.64	0.66	0.64	0.62	0.40	0.35	0.54	0.64	0.92	1.00

### Intra-diurnal variations in the atmospheric fungal population

**Fig 4:**
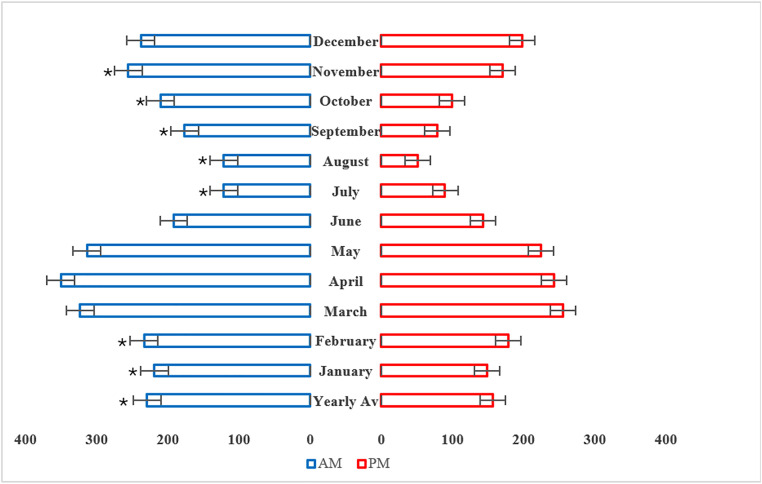
Differences in the atmospheric fungal load between the am and pm sampling periods over the study period. asterisk denotes statistical significance (p < 0.05).

Two samples per day were collected during the study period (in duplicate), during the morning (am) and afternoon (pm). The yearly average fungal count was significantly greater during the am period with statistical significance (P < 0.05), as shown in Fig 4. Statistically significant differences (p < 0.05) in fungal composition between the am and pm samples were also recorded in January, February, July, August, September, October and November (Fig 4). Additionally, the weighted Jaccard similarity coefficient index revealed 68% similarity between the am and pm samples in terms of atmospheric fungal distribution, as shown in Table 3.

**Table 3:** Similarity indices between intra-diurnal periods with respect to the daily fungal distribution. Similarity was determined through the weighted Jaccard similarity coefficient.

	AM	PM
AM	1.00	0.68
PM	0.68	1.00

### Meteorological interactions with fungal bioaerosols

Meteorological data were collected from Kuwait Meteorological Department, Directorate General of Civil Aviation. High temperatures (more than 40°C) were recorded during the period from May to September (Table 4). This heat is accompanied by low relative humidity ranging between 16.6% and 23.5%, as well as scarce rainfall, as shown in Table 4. Dust and haze were present throughout the year, especially during the period from April to September.

**Table 4:** Values of the monthly meteorological parameters of Kuwait City during the study period.

	Temperature (°C)			Relative Humidity (%)	Wind Speed (m/s)		Wind Gust (m/s)	Rainfall (mm)	Dust Storm (in days)	Blowing Dust (in days)	Haze (in days)	Dust Haze (in days)
	Min	Average	Max		Average	Max						
**January**	7.8	15.5	24.4	54.4	1.5	5.9	14.1	18.8	0.4	4.0	22.6	6.5
**February**	8.8	17.7	27.9	46.9	1.6	6.4	15.0	7.9	0.4	4.3	21.9	4.8
**March**	14.0	22.0	34.4	41.7	1.7	6.1	15.7	15.1	0.6	7.0	23.1	6.5
**April**	18.0	27.3	38.7	34.5	1.9	7.8	16.9	7.5	0.8	6.8	22.5	6.6
**May**	24.3	34.0	46.3	23.5	1.8	6.9	16.0	2.1	1.6	8.0	26.6	8.5
**June**	29.9	38.8	49.6	16.6	2.0	6.9	19.9	0.0	0.6	12.0	22.4	6.9
**July**	32.6	40.3	50.6	19.3	1.8	5.2	14.4	0.0	0.4	8.9	27.4	6.9
**August**	32.4	39.6	50.2	23.5	1.8	5.5	14.4	0.0	0.0	4.3	26.8	6.5
**September**	28.1	36.9	48.0	24.3	1.7	6.1	13.1	0.0	0.0	3.8	26.5	6.3
**October**	21.9	31.1	42.7	34.1	1.7	6.7	15.2	2.4	0.1	1.6	27.4	6.8
**November**	14.5	23.2	34.5	48.2	1.6	7.7	16.1	44.0	0.0	2.5	25.8	5.0
**December**	10.0	17.9	27.4	56.4	1.5	5.0	14.3	22.7	0.1	3.0	24.3	4.9
**Average**	20.2	28.7	39.6	35.3	1.7	6.4	15.4	10.0	0.4	5.5	24.8	6.3

**Fig 5:**
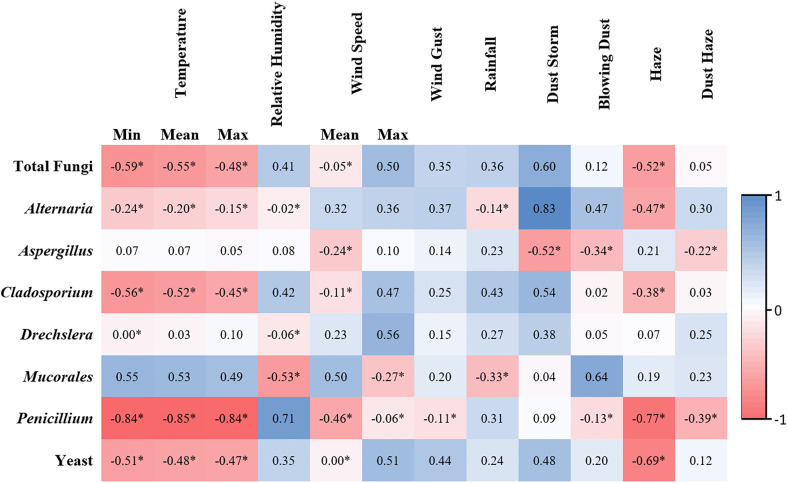
Correlations between meteorological data and captured fungal bioaerosols during the study period. Blue denotes a positive correlation, and red denotes a negative correlation. Values between 0.5 and 1 imply strong correlations, values between 0.3 and 0.49 imply moderate correlations, and values below 0.29 imply weak or no correlations. Asterisks indicate statistical significance (P < 0.05).

Pearson’s correlation coefficient was used to determine potential positive and negative correlations between fungal bioaerosols and meteorological parameters collected during the study period. A strong negative correlation was detected between most fungal genera and temperature (min, mean and max), as well as haze (Fig 5). Conversely, positive correlations were observed with wind speed, wind gust, rainfall and dust storms, as shown in Fig 5.

## Discussion

Aero-mycology is a branch of aerobiology that investigates the dynamics of airborne fungal bioaerosols in the atmosphere. These monitoring efforts aim to assist in realizing effective preventative measures to protect individuals with conditions like severe asthma, predisposing atopy and debilitating RT diseases.

In this study, fungal bioaerosols in Kuwait City were monitored from January 2016 to December 2023. The highest concentration recorded was in May 2018, reaching 577 CFU/m^3^, whereas the lowest concentration was 53 CFU/m^3^, recorded in August 2016 (Fig 1). Over the 8-year study period, the rates of fungal spores remained relatively high during the months of March, April and May, ranging from 269 to 296 CFU/m^3^, whereas in July and August, the lowest concentration was 86–106 CFU/m^3^, as shown in Table 1. Similar statistically significant monthly variations were reported in 1-year surveys of aeroallergens in Kuwait, Qatar, Saudi Arabia and Iran, where the lowest fungal bioaerosols were recorded during summer (June, July and August); however, the fungal count peak slightly differed from our findings, as they reported that the winter months December, January and February presented the highest airborne fungal count [4,13,22,23]. These differences can be attributed to the dedicated study period, as 1-year surveys may not provide accurate airborne fungal configurations unless sequential reporting is conducted. Additionally, different sampling methods were used in the aforementioned studies with different flow rates, device elevations and sampling durations, which may be the reason for the differences in the highest monthly fungal counts.

In this study, the most common fungal genera isolated were *Cladosporium*, *Aspergillus* and *Alternaria*, with prevalence rates of 49%, 16% and 14%, respectively, as shown in Fig 2. Similar fungal genera were reported to be common in previous local studies as well as in regional surveys in Kuwait, Saudi Arabia, Iran, Qatar and Jordan [13,24–28]. This consistent fungal distribution may be because these particular fungal genera are abundant in soil throughout the region and are thought to be carried during wind action and dust events [14,17]. Moreover, certain fungal bioaerosols were statistically significant in certain months of the year, which may suggest a seasonal distribution of some fungal genera (Fig 3). Indeed, *Alternaria* and *Cladosporium* prevalence was statistically significant during cold and moderate months, as shown in Fig 3. However, some genera exceeded that range into hot months, which can be attributed to their innate thermotolerance [29,30].

A similar finding was reported regionally, as *Aspergillus* and *Cladosporium* were significantly detected during similar periods (March to July) [14]. Moreover, fungal seasonal patterns have also been reported in Kuwait through the analysis of immunological markers of *Aspergillus*, *Alternaria* and *Cladosporium* in individuals with asthma; however, they reported a different prominence period (September to November) [4]. Conversely, other regional studies suggested no fungal seasonal patterns but rather a somewhat homogeneous distribution of fungal bioaerosols throughout the year [31]. This homogenous distribution is elucidated to be due to the presence of these fungal genera in the soil, which functions as a reservoir for their atmospheric distribution all in all, these contradicting findings need further elucidation [31].

Moreover, the monthly similarity analysis revealed two periods in which the airborne fungal distributions were similar. The highest Jaccard similarity coefficient score for atmospheric fungal composition was recorded between March, April and May and between November and December (Table 2). These months are considered transitional periods between the two main seasons in Kuwait (summer and winter), where the weather is stormy and often unpredictable with high wind speed and dust dispersal [15]. Unfortunately, we could not find related regional reports for comparative analyses.

Fungal bioaerosol concentrations during different intra-diurnal periods (am and pm) were analyzed in this study. The yearly average fungal count was significantly greater during the am period with statistical significance (P < 0.05), as shown in Fig 4. Statistically significant differences (p < 0.05) in fungal composition between the am and pm samples were also recorded in most monthly analyses (Fig 4). The statistically significant increase in airborne fungi during the am period may be attributed to the lack of solar radiation, the reduction in ambient temperature and the increase in relative humidity during the night; all these factors may result in increased atmospheric conidia detection [32]. Additionally, the increase in wind action after sunrise promotes the detachment of fungal conidia and their subsequent release into the atmosphere [33,34]. Notably, the composition of fungal bioaerosols between intra-diurnal periods was not highly similar, as shown in Table 3. Consistent with our findings, a lower similarity coefficient was reported in Qatar at 48% between 6:00 am and 12:00 pm [13]. The minimal similarity in intra-diurnal periodicity of airborne fungal composition reported in this study may be attributed to the pattern of fungal spore discharge mechanisms in relation to external factors such as temperature and humidity [33]. For example, *Cladosporium* conidia are formed in loose chains that require reduced humidity to prompt the collapse of conidial chains and subsequent release into the atmosphere; therefore, a relatively high concentration of *Cladosporium* spores in the afternoon is generally expected [6]. However, Basidiomycota spores are often detected in the late night to early morning period because of their dependency on molecular water pressure, which facilitates spore emission [6].

The meteorological data obtained in this study revealed several expected results, such as high mean temperatures during the summer and moderate mean temperatures during the winter months, as shown in Table 4. The average yearly mean temperature was 28.7°C, which is similar to the previously reported mean temperature in Kuwait from 2001 to 2017 of 29°C; however, it remains well above the regional mean temperature, which is 23.4°C [35]. However, the average relative humidity (35.4%) has increased over that previously reported figures in Kuwait at 27%, yet it remains in tune with the average regional rate [35]. The average wind speed (1.7 m/s) was lower than the previously reported rate in Kuwait at 2.58 m/s [35]. Moreover, dusty weather phenotypes during the study period were identified at an average of 147 days per year, which is less than the previously reported rate in Kuwait of 255 days per year [15,36]. In particular, the sum of dust storms recorded in this study was 40 events, averaging 5 storms per year (data not shown), whereas from 2001 to 2017, the sum of dust storms was greater at 131 events, averaging 8.2 per year [35]. The major sources of dusty weather in Kuwait include the drained marsh area in southern Iraq, dry marshes in Iran and the northeastern desert of Saudi Arabia; thus, efforts to increase the vegetation cover in these areas as well as within Kuwaiti borders may have resulted in this favorable reduction [36]. Additionally, the lower average wind speed reported in this study may have been implicated in this reduction, as wind speed affects the long-distance transport of particulate matter in the atmosphere [35]. Notably, the variation in study design and instruments between our study and previous reports may be responsible for these discrepancies or could involve uncharacterized biological and geological factors; all in all, further investigations are needed.

Pearson’s correlation coefficient was used to determine positive and negative correlations between fungal bioaerosols in the atmosphere and various meteorological parameters, such as temperature, relative humidity, wind speed, rainfall and dusty weather phenotypes (Fig 5). The total fungal count and most fungal genera identified in this study were significantly negatively correlated with temperature (p < 0.05). A negative correlation between the rate of airborne fungal bioaerosols and elevated temperatures has been reported before in Qatar, Saudi Arabia, Iran and Pakistan, as high temperatures accompanied by solar radiation effectively destroy soil-borne and airborne fungal bioaerosols [8,13,22,23]. Interestingly, in climates with temperatures that are well below the mesophilic temperature range (20–45°C), opposite findings may be exhibited, as low temperatures may limit the metabolic activities of microorganisms; thus, a positive correlation between temperature and the rate of fungal bioaerosols may be reported [37,38].

Moreover, there was a positive correlation between the rate of fungal bioaerosols and relative humidity, wind speed and rainfall, but this correlation was not statistically significant. Similar findings were reported in Kuwait, Qatar, Iran, and Turkey [9,11,13,14]. Indeed, moisture in the atmosphere and moderate wind action may function as fungal growth factors and high dispersal forces, respectively, which may explain the positive correlations reported in this study [37,39].

Dust phenotypes are common weather phenomena in Arabia, as wind action carries particulate matter resulting from rocky formations and soil structure erosion, especially in areas that are arid and lack vegetation cover [15]. In this study, a positive correlation was detected between fungal bioaerosols in the atmosphere and dusty weather phenotypes (dust storms, blowing dust and dust haze). It has been suggested that dust not only acts as a transport vehicle for fungal pathogens but also lowers the ambient temperature, which may provide a more hospitable climate for conidial germination and spread [17].

Interestingly, temperature and relative humidity were not correlated with atmospheric *Aspergillus* rates, which was confirmed in a report from Qatar [13]. The small conidial size along with the innate thermotolerance and hygroscopic properties of *Aspergillus* allow it to withstand elevations in temperature and atmospheric moisture [29,40]. Indeed, the global ubiquity of *Aspergillus* is driven by survivability attributes that protect against eco-physiological and bio-physical challenges [41]. In fact, several reports demonstrated the ability of *Aspergillus* to withstand exposure to extreme temperatures that reach 85°C as well as extreme limitation of water activity at less than 0.6 [42–44]. Thus, it is understandable that no correlation with temperature and humidity were demonstrated in this study. Similar findings were observed for *Drechslera* rates; however, owing to the relatively lower prevalence, we cannot suggest similar postulations.

Haze is characterized by the suspension of fine particulate matter in the atmosphere, e.g., sulfur dioxide (SO_2_), nitrogen dioxide (NO_2_), and carbon monoxide (CO), thus creating stagnant atmospheric conditions, especially in flat terrain countries such as Kuwait [45]. Our findings revealed a negative correlation between fungal bioaerosol rates and haze events, as shown in Fig 5. Several studies from China, Turkey and Poland have reported similar findings [46–48]. It has been postulated that during haze events, suspended chemicals in the atmosphere may modify fungal conidia morphology, thus reducing their aerodynamic properties [49,50]. Additionally, suspended inorganic ions (Cl^-^, Ca^2+^, Na^+^ and K^+^) and heavy metals (Pb, As and Zn) may disrupt the structure and function of microorganisms and may impose oxidative stress via the generation of reactive oxygen species [51–53]. However, other reports concluded that microbial aerosols during hazy events are increased by 2–5-fold when compared with those on clear days, thus illustrating a clear need for further investigations [34,54,55].

## Conclusion

This study examined the dynamics of fungal bioaerosols in the atmosphere of Kuwait City, with respect to fungal configuration, distribution, intra-diurnal variations and correlations with meteorological parameters. Through the collection of data during the 8-year study period, we determined that *Cladosporium*, *Aspergillus* and *Alternaria* were the most common genera in the atmosphere. Additionally, fungal bioaerosols in the atmosphere exhibit seasonal patterns and intra-diurnal change in prevalence. Two consistent periods during the year presented similar fungal compositions: March–April–May and November–December. In this study, a negative correlation was detected between airborne fungal bioaerosols and temperature, whereas a positive correlation was detected with relative humidity, rainfall, wind speed and dust. It is worth noting that the findings presented in this study may not reflect the dynamics of indoor fungal bioaerosols.

There is a regional dearth of data on rates of fungal bioaerosols. Indeed, collaborative regional research is needed to advance our understanding of the implications of elevated rates of fungal bioaerosols and what may be suitable means of mitigation. Efforts such as this study will hopefully help facilitate regionally specific air quality management decisions, provide baseline information for allergists and other health-care providers and improve health-burden estimates of bioaerosol exposure to make meaningful risk assessments.

### Importance

Respirable fungal allergens have a negative impact on predisposed atopic patients with asthma; increased exacerbation episodes and associated mortality burden are some of the related challenges facing healthcare providers. Aero-mycological studies provide a baseline understanding of fungal dynamics and assist in realizing effective preventative measures to protect predisposed individuals. In a predominantly dusty atmosphere, airborne fungal spores play a significant role in complicating the symptoms of predisposed atopic patients. In this study, we identified the monthly prevalence and associations of the most common airborne fungal allergens in Kuwait City. We also identified the meteorological parameters associated with high airborne fungal loads. We also demonstrated seasonal patterns of outdoor fungal bioaerosols in the atmosphere of Kuwait City. Surveys such as this aim to inspire future work to assess the implications of such aerobiological data for public health.

## Supporting information

S1 File Dust Phenotypes recorded during the period (2016–2023). (XLS)

S2 File Rainfall measurements from Kuwait Airport Station. (XLS)

S3 File Temperature, relative humidity and wind speed recorded during the period (2016–2023). (XLSX)
